# Slug Flow Coprecipitation Synthesis of Uniformly-Sized Oxalate Precursor Microparticles for Improved Reproducibility and Tap Density of Li(Ni_0.8_Co_0.1_Mn_0.1_)O_2_ Cathode Materials

**DOI:** 10.1021/acsaem.2c03563

**Published:** 2023-03-06

**Authors:** Mingyao Mou, Arjun Patel, Sourav Mallick, K. Jayanthi, Xiao-Guang Sun, Mariappan Parans Paranthaman, Sophie Kothe, Ena Baral, Selma Saleh, Jethrine H. Mugumya, Michael L. Rasche, Ram B. Gupta, Herman Lopez, Mo Jiang

**Affiliations:** † Department of Chemical and Life Science Engineering, Virginia Commonwealth University, Richmond, Virginia 23219, United States; ‡ Chemical Sciences Division, Oak Ridge National Laboratory, Oak Ridge, Tennessee 37831, United States; § Ionblox Inc., Fremont, California 94538, United States

**Keywords:** lithium-ion battery, NCM811, tap density, coprecipitation, slug flow

## Abstract

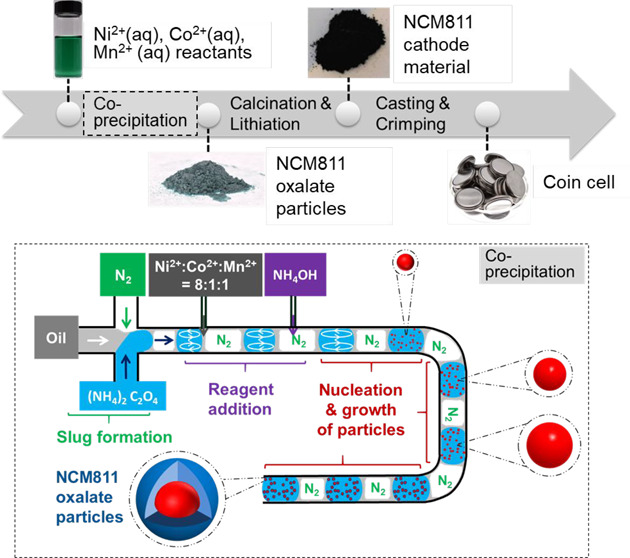

The microparticle quality and reproducibility of Li(Ni_0.8_Co_0.1_Mn_0.1_)O_2_ (NCM811) cathode materials are important for Li-ion battery performance but can be challenging to control directly from synthesis. Here, a scalable reproducible synthesis process is designed based on slug flow to rapidly generate uniform micron-size spherical-shape NCM oxalate precursor microparticles at 25–34 °C. The whole process takes only 10 min, from solution mixing to precursor microparticle generation, without needing aging that typically takes hours. These oxalate precursors are convertible to spherical-shape NCM811 oxide microparticles, through a preliminary design of low heating rates (e.g., 0.1 and 0.8 °C/min) for calcination and lithiation. The outcome oxide cathode particles also demonstrate improved tap density (e.g., 2.4 g mL^–1^ for NCM811) and good specific capacity (202 mAh g^–1^ at 0.1 C) in coin cells and reasonably good cycling performance with LiF coating.

## 1 Introduction

The expanding demands of lithium ion batteries, in portable electronic devices (e.g., smartphones, tablet PCs) and environmental-friendly vehicles (e.g., electric and/or hybrid vehicles), require a further increase in reproducibility and electrochemical performance (e.g., specific capacity, tap density) and a reduction in cost.^1−10^ The cathode material is one of the key cost drivers in batteries, with layered nickel rich LiNi_*x*_Co_*y*_Mn_*z*_O_2_ (NCM*xyz*) cathode as one widely used material.^11,12^ The composition, phase purity, morphology, and size of the NCM material microparticles directly affect battery performance.^13−18^

There are many synthesis methods^19^ for these NCM material microparticles, including coprecipitation, spray drying/pyrolysis,^19,20^ solid-state methods, sol–gel synthesis,^21^ and combustion methods.^22^ Solid-state-based methods are straightforward, but the coprecipitation method provides more uniformity in mixing, with better product control and simpler equipment needs; thus, it is popular in industry. For Li(Ni_0.8_Co_0.1_Mn_0.1_)O_2_ (NCM811)-based materials, most existing coprecipitation synthesis routes (Table 1) take more than 10 h (including aging time) of hydroxide reactions^23−27^ in stirred-tank reactors, with temperature (50–65 °C), pH, and stirring tuned to control the product quality.^13,14,16,28,29^ Besides these hydroxide reactions, a few recent studies take a shorter time to synthesize NCM811 materials with very good electrochemical performance, by using oxalate coprecipitation reactions in hydrothermal reactors (up to 100 °C), without needing pH control.^30^

**Table 1:** Representative Coprecipitation Synthesis Reaction Conditions of NCM811-Based Cathode Materials and Corresponding Tap Density and Specific Capacity

NCM811-based materials	precursor precipitation synthesis reaction	precursor synthesis temperature (°C)	total precipitation time (h)	tap density (g mL^–1^)	voltage window (V vs Li/Li^+^)	specific capacity of NCM811-based material (mAh g^–1^)	ref
NCM811	Hydroxide	65	12	2.015	2.9–4.3	185.04 (0.1 C) (1 C = 190 mA g^–1^)	(23)
	Hydroxide	50	28	N/A	2.7–4.3	177 (0.1 C) (1 C = 190 mA g^–1^)	(25)
	Hydroxide	55	N/A	N/A	2.7–4.3	180 (0.2 C) (1 C = 180 mA g^–1^)	(26)
	Hydroxide	50	36	2.26	2.3–4.3	198.3 (0.1 C)	(27)
	Oxalate	100/125–room T	1 (room T) + 0.3 (100 °C) + gradual heating and cooling time	N/A	2.8–4.3	183/188.5 (1 C) (1 C = 180 mA g^–1^)	(30)
	Oxalate	25–34	0.1–0.2	2.4 ± 0.1	2.8–4.3	202.0 (0.1 C) (1 C = 200 mA g^–1^)	this work
Mg-substituted NCM	Hydroxide	65	12	1.95–2.10	N/A	184.2–191.8 (0.1 C) (1 C = 190 mA g^–1^)	(23)
B-doped NCM811	Hydroxide	60	12	N/A	3.0–4.3	163 (0.04 C) (1 C = 160 mA g^–1^)	(24)
Co and Mn coated NCM811	Hydroxide	50	28	N/A	2.7–4.3	180 (0.1 C) (1 C = 190 mA g^–1^)	(25)
NCM811 with concentration gradient	Hydroxide	50	35	2.38	2.3–4.3	198.7 (0.1 C)	(27)

All these NCM811 precursor reactors are based on tank geometry. Conventional stirred-tank reactors or crystallizers have been thoroughly analyzed and compared to advanced flow reactors or crystallizers on other molecules including fine chemicals and pharmaceuticals,^33−41^ such as on product crystal variability and reproducibility and equipment scale-up strategy.^42^ For various NCM precursor synthesis with hydroxide reactions, recently running stirred tanks in the continuous mode (instead of the batch mode) were shown to improve particle uniformity,^31,32^ although the intrinsic limits of tanks still remain. For low-cobalt NCM811 precursor synthesis, more and deeper investigations on the promising approach/process with fast oxalate coprecipitation reaction are needed, especially that facilitate reproducible and facile reactions (e.g., low temperature, simple equipment needs) and convenient scale up.

This Article presents a coprecipitation process/reactor that directly generates uniformly-sized spherical precursor microparticles for NCM811, based on the oxalate coprecipitation reaction even at room temperature, with tunable productivity using the same equipment. The process and equipment (inexpensive disposable tubing) for reaction precipitation are designed based on scalable tubular slug flow, which is already utilized in tunable crystallization of organic molecules, such as amino acids and pharmaceuticals.^43−46^ For these new oxalate precursor particles from slug flow, heating rates of the calcination and lithiation processes are designed toward maintaining their spherical morphology. The material tap density and specific capacity of the lithiated oxide microparticles are characterized in coin cells and compared with a literature value from stirred tank reactors (Table 1).

## 2 Experimental Methods

### 2.1 Materials

Nickel sulfate hexahydrate (≥98%), cobalt sulfate heptahydrate (≥99%), manganese sulfate monohydrate (≥99%), ammonium oxalate (≥99%), ammonium hydroxide (28% NH_3_ in H_2_O, purity ≥99.99%), light mineral oil, and lithium hydroxide (≥98%) were purchased from Sigma-Aldrich. All chemicals were used as received without further treatment. All the solutions were prepared with deionized water.

### 2.2 Coprecipitation Synthesis of Precursor Microparticles in Tank Reactors

A coprecipitation reaction (eq 1) to synthesize precursor (N_*x*_C_*y*_M_1–*x*–*y*_)C_2_O_4_ (0.3 ≤ *x* ≤ 0.9, 0 ≤ *y* ≤ 0.3, for cobalt-free material, *y* = 0) is modified as follows:^47^ stoichiometric amounts of nickel(II) sulfate hexahydrate, cobalt(II) sulfate heptahydrate, and manganese(II) sulfate monohydrate were dissolved in deionized water.
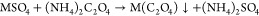
1where M = Ni, Mn, and/or Co mixture. The solution was transferred into a reactor under a nitrogen atmosphere and at a 25–34 °C range of temperature. An aqueous solution of ammonium oxalate was added to the reactor as a chelating reagent and precipitation reagent, and ammonium hydroxide was added to adjust pH to 8.5. The precipitate was filtered, washed, and then dried at 60 °C. Various reactor configurations are applied for coprecipitation synthesis, at the same reaction conditions, including reactant concentrations and volumes, pH, and residence time. The *stirred tank reactors* (either batch or semibatch modes) are regular round-bottom flasks.

**Figure 1:**
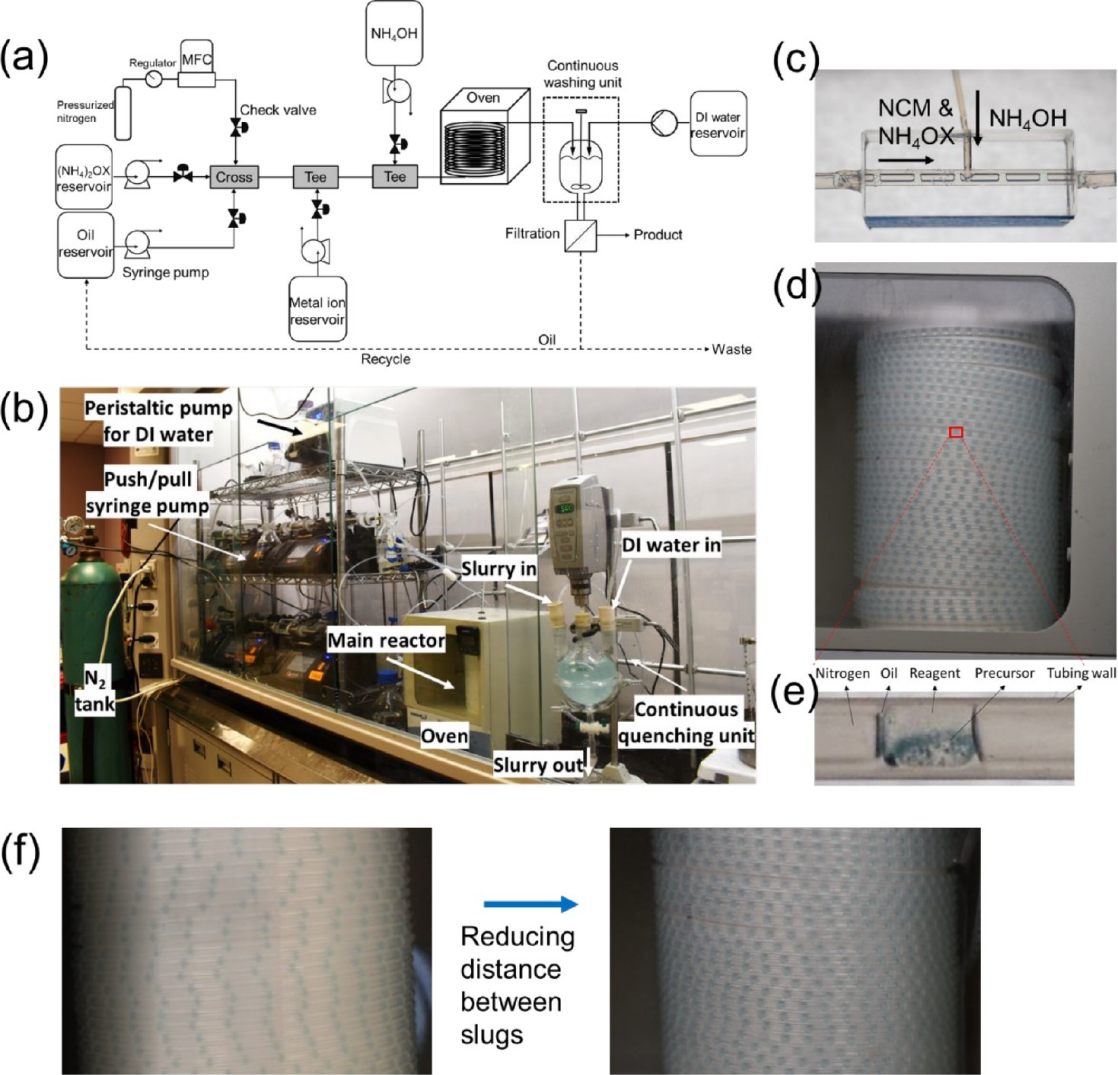
(a) The process flow diagram and (b) a photo of the slug flow reactor for oxalate precursor synthesis. Photo of (c) the reactant mixing Tee-connector (home-built) and (d) of the slug flow reactor (with tubing for slug flowing inside wrapped around a cylinder). (e) Zoom-in photo of a representative slurry slug including products (blue), with oil layer for reducing fouling; (f) an example of scaling up slug flow reaction without changing equipment, by reducing distances between adjacent slugs (green color).

The *slug flow reactor* (Figure 1a,b) consists of four syringe pumps; one gas mass flow controller; one heating zone; one continuous washing unit; one filtration system; fluorinated ethylene propylene (FEP) tubing with an inner diameter of 2.4 mm (Figure 1c,d,e). Push/pull autofill syringe pumps (Harvard Apparatus, Model#703009) along with a continuous delivery valve box (Harvard Apparatus, model# 557013) were used to feed liquid with zero shutdown time. All the solutions are transferred into the reservoir bottle, and the syringe pump can dispense them continuously until the target scale. A mass flow controller (MFC, Omega, model# FMA-2716A) was connected to the nitrogen tank to infuse gas. A three-phase liquid/liquid/gas slug flow-based reactor (3PSFR) was designed for this process to minimize fouling on the tubing wall (Figure S1). Oil serves as the carrier phase isolating chemical reagents from the tubing. Nitrogen is the inert spacer gas that creates boundaries to enhance mixing inside the liquid slug without an external mixer. At the first cross mixer, oil, nitrogen, and ammonium oxalate were infused into the three inlets to form a three-phase slug flow inside the FEP tubing. Translucent FEP tubing was selected to visualize the process and due to its high tolerance to a wide range of temperatures and chemicals. All the inlets were equipped with a check valve to prevent backflow. Then, NCM metal ion solution and ammonium hydroxide was sequentially injected into the ammonium oxalate slug via a house-built Tee connector each. The outlet of the slug flow reactor was directly connected to a three-neck flask with drainage for quenching the reaction. The quenching flask was filled with ca. 400 mL of water prior to the collection of slurry slugs. During the collection, the drainage valve is open and connected to the filtration system, and fresh DI water is continuously fed into the flask for quenching and washing. Flow rates of the drainage and the DI water are maintained the same. The slurry was immediately filtered, washed, and dried.

### 2.3 Lithiation of NCM811 Oxalate

Toward composition of LiN_0.8_C_0.1_M_0.1_O_2_ (NCM811), the NCM811 oxalate precursor was mixed with LiOH·H_2_O (1:1.05) in a mortar and pestle for 10 min. The mixture was then subjected to calcination at a high temperature of 850 °C under continuous oxygen flow. The following heating rates are maintained during raising the temperature: (i) 0.1 °C min^–1^ was maintained from room temperature to 500 °C, (ii) 0.8 °C min^–1^ was maintained from 500 to 850 °C, and (iii) 850 °C was maintained up to 12 h. Finally, the as-obtained, black-colored product (NCM811) was collected and crushed well.

### 2.4 Composition and Morphology Characterization of Microparticles

The morphology and element distribution of the precursor or lithiated powders was checked with scanning electron microscopy (SEM, SU-700, Hitachi) equipped with an energy dispersive spectroscopy (EDS) system. Focused ion beam scanning electron microscope (FIB-SEM, Carl Zeiss Auriga) featuring a Schottky field emission Gemini electron column was used to cut the particles. The crystalline form was determined by X-ray powder diffraction (PANalytical, Empyrean X-ray diffractometer) using a Cu Kα radiation source. The composition of the synthesized powders was measured by inductively coupled plasma-optical emission spectrometry (ICP-OES, Agilent Technologies, 5110-MS). Tap density was measured using Autotap from Quantachrome instruments. Thermogravimetric analysis (TGA) and differential scanning calorimetry (DSC) thermal graphs were obtained from TGA Q500 and DSC Q1000 (TA Instruments) at a ramp of 10 °C min^–1^. Crystal size was analyzed by a laser diffraction instrument (Sympatec HELOS equipped with ASPIROS feeder) operating at 300 kPa.

### 2.5 Electrochemical Test of Li(Ni_0.8_Co_0.1_Mn_0.1_)O_2_ Microparticles

The cathode was prepared by coating the aluminum foil with a slurry, containing NCM811, carbon black and polyvinylidene difluoride (PVDF) in *N*-methyl pyrrolidone (NMP) [weight ratio of 8:1:1] by a doctor blade technique followed by drying at 130 °C for 12 h under vacuum. The cathode was then calendared and cut to a diameter of 15 mm. The loading of the active material on the cathode was 8.5–9.5 mg cm^–2^ and the calendaring thickness was 110 μm (including 15 μm thick Al foil). The coin cells were fabricated by placing the electrolyte-soaked Celgard 2340 Trilayer Microporous membrane (separator) in between the cathode (NCM811-coated Al foil) and anode (lithium metal foil) inside an argon-filled glovebox. 1.0 M LiPF_6_ in ethylene carbonate/dimethyl carbonate (EC/DMC = 50/50 (v/v)) was used as the electrolyte. The electrochemical performance of the cathode was evaluated through cyclic voltammetry and charge–discharge in a battery cyclers (BTS8-MA from MTI Incorporation, and SYS-LBT20084 from Arbin Instrumentation) considering 1 C = 200 mA g^–1^. The electrochemical impedance spectroscopy (EIS) was performed at various charging and cycling states with an amplitude of 5 mV within a frequency range of 100 kHz to 10 mHz using a Gamry potentiostat Interface 5000E. All electrochemical tests were conducted at room temperature.

## 3 Results and Discussion

### 3.1 Fast Coprecipitation Synthesis of Uniform-Size “Core-Shell” Oxalate Precursor Microparticles

**Figure 2:**
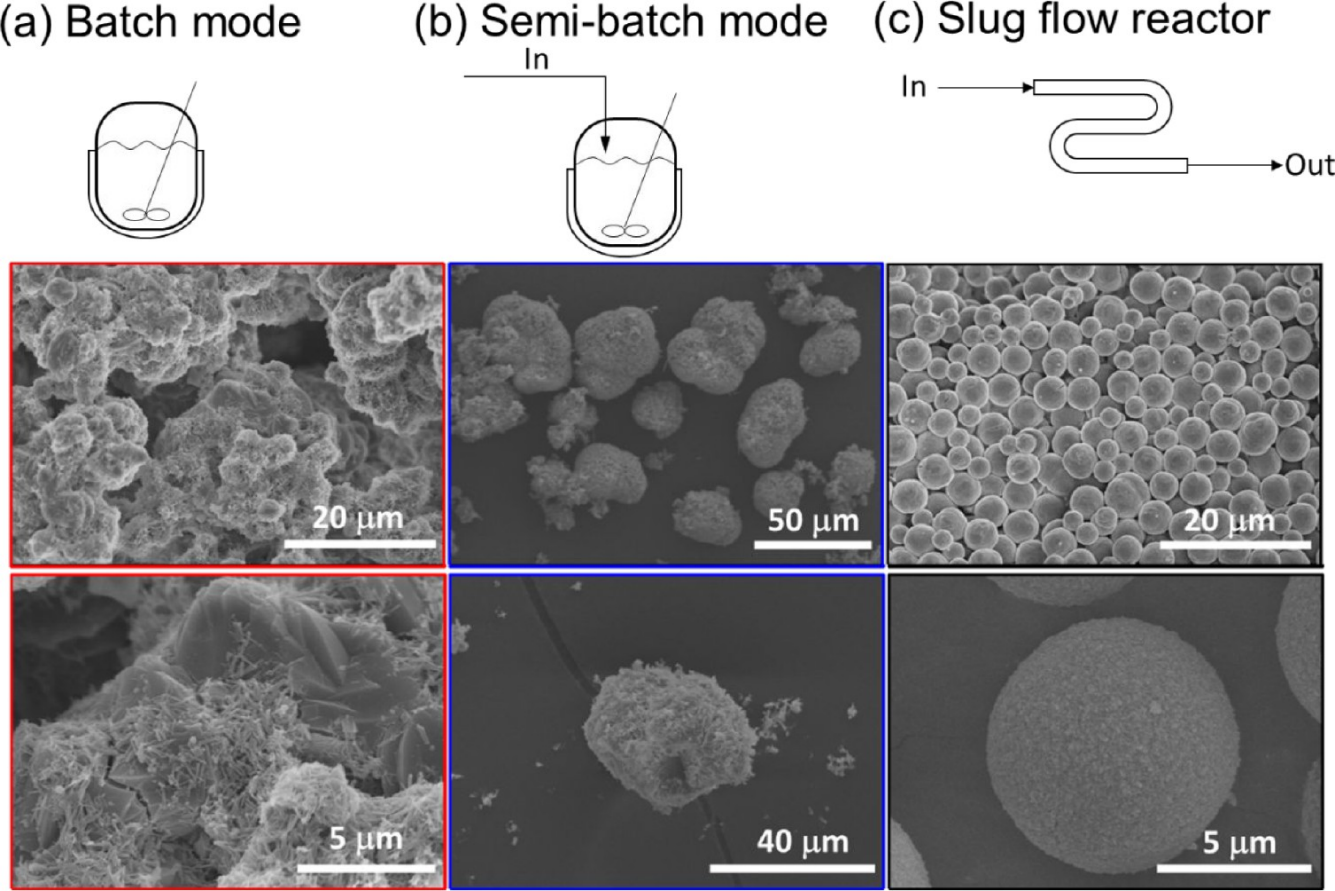
SEM image of NCM811 oxalate precursor microparticles synthesized from different reactor configurations. Columns (a) batch mode and (b) semibatch mode, both for stirred-tank reactors, with reactants adding once or gradually. Column (c) slug flow reactor in Figure 1.

Uniform-sized slugs containing aqueous solutions (separated by nitrogen, Figure 1a,b,d) serve as a series of individual milli-fluidic reactors for coprecipitation synthesis. As in Figure 2, water-nitrogen slug flow reactors allow synthesis of more uniform-sized microparticles (also with less aggregation, Figure S2) than conventional stirred-tank-based reactors, even at the same reaction conditions, such as reactant concentrations, temperature (25–34 °C), and pH (8.5). The outcome particle uniformity likely comes from a spatially uniform reaction environment, from the slug flow properties of internal recirculation and large surface area-to-volume ratios, for enhanced heat and mass transfer.^43,44,48−52^ The large surface area-to-volume ratio for tubular processes typically increase the chance of fouling (Figure S1), and here, we introduce a third phase of oil between aqueous slugs and tubing wall that minimizes fouling (Figure 1e), even for running the reaction continuously for multiple hours.

**Figure 3:**
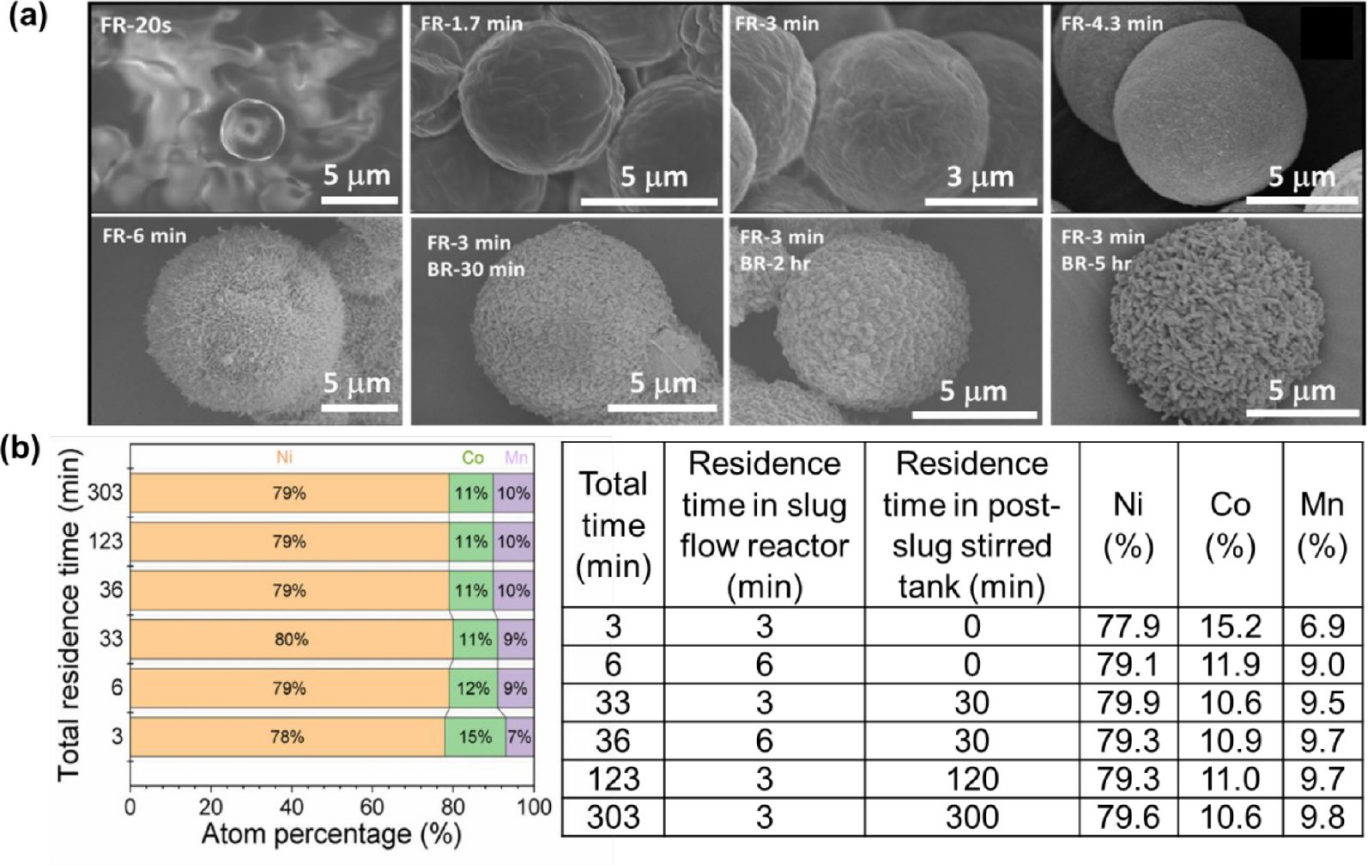
(a) SEM images of NCM811 oxalate precursor synthesized at different reaction times, directly from the slug flow reactor (FR), and/or with postslug flow ripening/aging in the stirred-tank at the batch mode (BR). (b) Composition comparison of oxalate precursors (in (a)) at different reaction residence times.

Within 6 min (Figure 3a,b) of coprecipitation reaction in slug flow, oxalate precursor microparticles of proper composition and 5-μm size are synthesized. The reaction time in slug flow (on the order of minutes) is much shorter than typical similar processes in stirred tanks (Table 1, 10 h or more). One possible reason is that long-time ripening is typically needed for improving size uniformity of microparticles from stirred tanks, but here, the microparticle sizes from slug flow are already uniform within 6 min; thus, there is no need for postreaction ripening. The uniform size distribution of crystals likely come from a spatially uniform reaction and crystallization environment (e.g., reactant concentrations, temperature) from slug flow. Specifically, slug flow allows the reduction of a larger volume of continuous liquid down to a large number of smaller-volume individual slugs of uniform sizes (e.g., Figure 1c,d,e, ∼5 mm size in each dimension),^43−46^ and each slug has much better heat and mass transfer than a larger tank/flask, due to both the smaller volume and intrinsic recirculation flow.^43−46^

In addition, even with 2 h of ripening (postslug flow, Figure 2a,b), the microparticles from slug flow do not increase size evidently (also solid mass remains similar), indicating most reactions have already been completed within the short time. The composition of these uniform microparticles could also be tuned within a range (e.g., 7–10% Mn here in Figure 3b) by adjusting the reaction residence time in the slug flow reactor.

**Figure 4:**
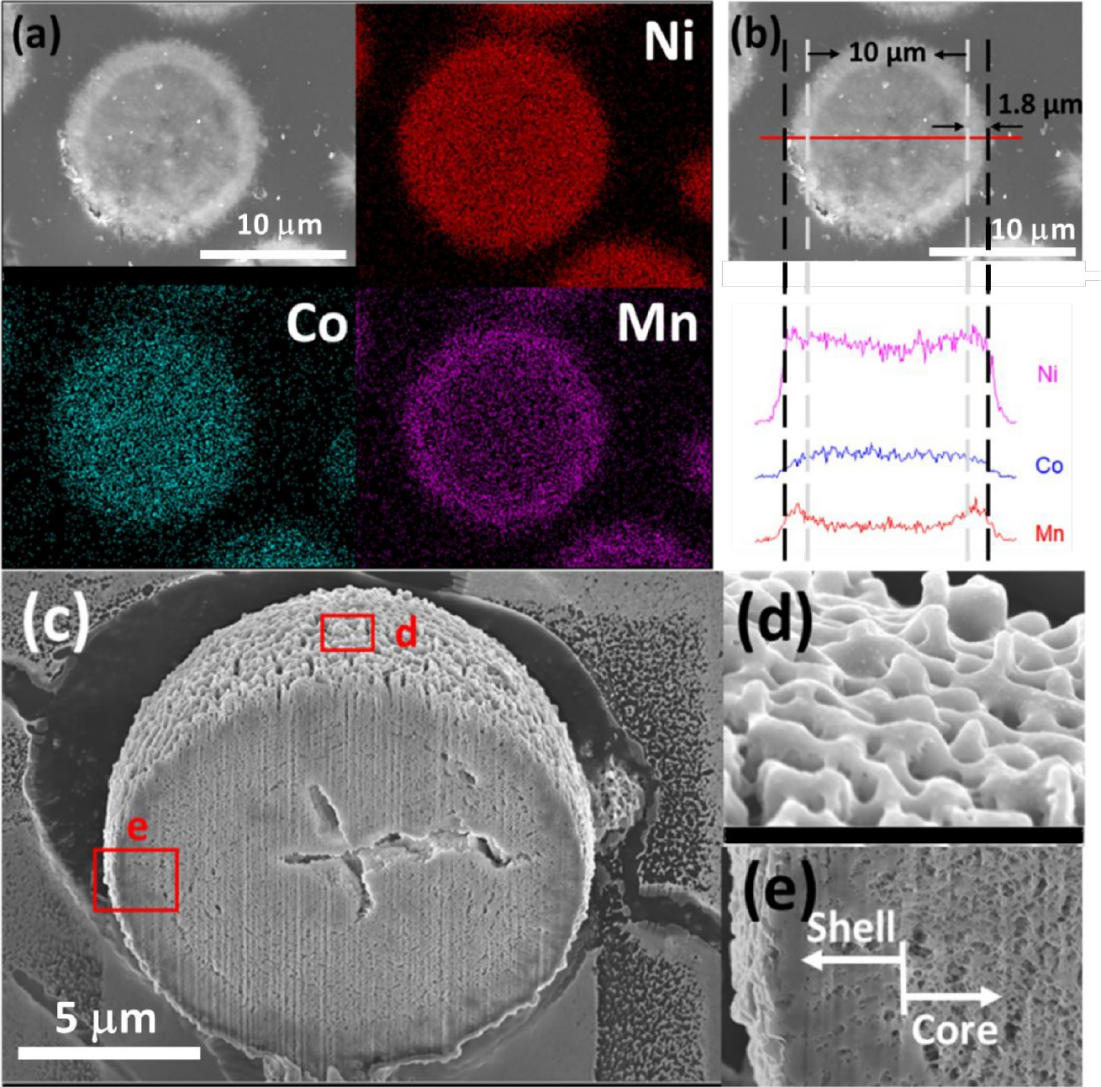
Element mapping of (a) the whole cross-section and (b) line scanning (red horizontal line) for NCM811 oxalate precursor microparticles (from Figure 3a, FR 6 min and BR 30 min). The outer surfaces for the shell and core of the microparticles are indicated with a black and gray dashed line, respectively. FIB-SEM images of the NCM811 oxalate precursor microparticles in (a, b) showing (c) detailed cross-section, (d) particle surface, and (e) particle core–shell boundary.

**Figure 5:**
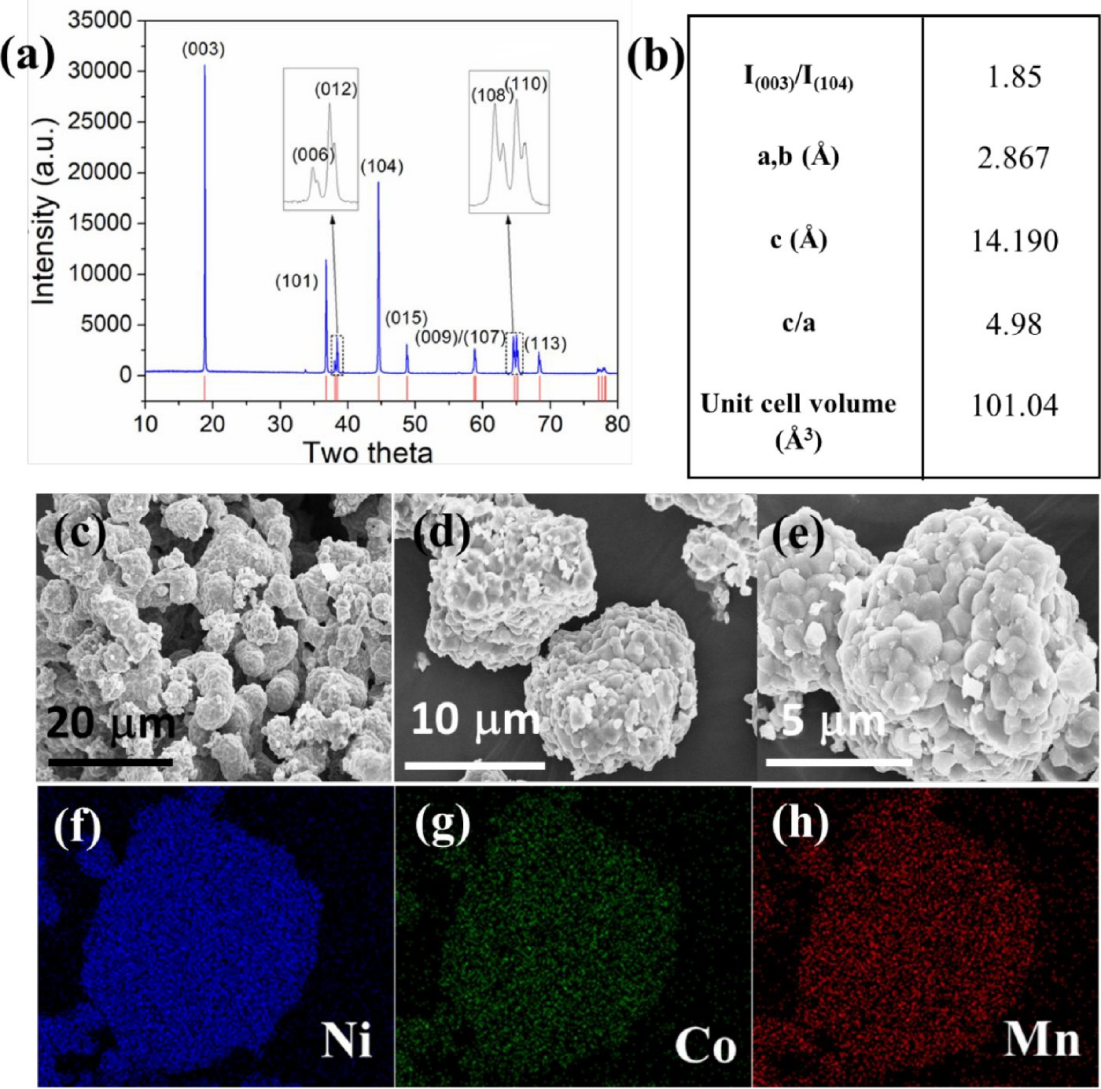
(a) XRD pattern and (b) crystal parameters (from Rietveld refinement) of the calcinated/lithiated NCM811 cathode material (before electrochemical testing). The red lines are reference spectra from ICDD# 04-023-5128. The crystal parameters postcycling are in Table S1. (c–e) SEM images and (f–h) elemental mapping of the NCM811 materials.

The element radial profile of the oxalate microparticles (Figure 4a,b) shows that manganese and nickel are richer in the shell than the core and cobalt is richer in the core than the shell. One possible reason for this radial profile is the difference in oxalate precipitation kinetics of each individual metal ion from preliminary experiments (data not shown). This “core-shell” structure of the oxalate microparticles is further shown in the cross-sectional SEM images (Figure 4c,d,e). As a side comment, the current core–shell distribution of 3 metals (Figure 4) is not the same as the designed profile in previous reports.^14^ (Manganese and cobalt are richer in the shell than the core, and nickel is richer in the core than the shell.) In both cases, manganese is richer in the shell and considered to have improved thermal and mechanical stability.^53,54^ A topic of future study is to draw a correlation between the current core–shell structure with NCM performance, once the issues are solved for core–shell structure loss during calcination (Figure 5f,g,h), and microcracking during cycling. As discussed in the next section, both phenomena are not uncommon for high-Ni cathode materials.

The microparticle size/morphology reproducibility has been confirmed by reaction at different scales, from 1 to 100 g of solid microparticles. While most processes need additional experiments and troubleshooting in larger scale equipment (e.g., larger diameters for reactor tanks or tubing) for scaling up, slug flow reactors (with stable uniform slugs formed and fouling minimized^43^) can scale up using the same equipment, at a constant microparticle production rate of 6–7 g h^–1^. This scale up is realized by increasing the slug number per unit time or space with 2 methods: (1) by simply running the experiment for a longer time with more slugs^43,46^ (e.g., continuously feeding reactants and collecting products from 6 min to 6 h) and/or (2) by increasing the number of slugs per unit time, such as through reducing the slug-to-slug distance (Figure 1f).

### 3.2 Oxalate Calcination/Lithiation Process Design for Retaining Morphology and Improving Tap Density of NCM811

The design goal of the calcination and lithiation process is to maintain the spherical morphology of the oxalate precursors during heating. In this study, no pre-calcination (including cooling between pre-calcination and calcination) was used, but the calcination step is decoupled into two stages of heating (at two different heating rates). The mixture of LiOH and oxalate precursors is first heated from room temperature to 500 °C at a constant slow rate of 0.1 °C/min to remove structural water and gas phase from oxalates. 500 °C is chosen based on typical pre-calcination temperature and LiOH decomposition temperature^55^ and DSC and TGA in Figure S3. After the mixture reaches 500 °C, it is heated at a constant regular rate of 0.8 °C min^–1^ from 500 to 850 °C and then held at 850 °C for 12 h to complete the lithiation process and achieve high crystallinity. Our preliminary test and observation (data not included) also shows that a constant heating rate of 1 °C/min for calcination (similar to the heating rate in common experiments using hydroxide as precursors) from room temperature to 850 °C will destroy the spherical morphology of oxalate precursors. Considering oxalate precursors were only recently used for NCM811 synthesis (Table 1), the corresponding calcination process and heating rates are going to be improved in the future.

The XRD pattern of the lithiated oxalate (Figure 5a) indicates the phase-pure NCM811 formed with a hexagonal α-NaFeO_2_ type structure and the space group of (*R*3*m*). The well-ordered layered structure is proved by the distinct splitting of (006)/(012) and (108)/(110) peaks.^24,56^ The crystal parameters and the *c*/*a* ratio of 4.98 in Figure 5b further indicates good hexagonal structure of the as-synthesized NCM811.^56,57^ SEM images (Figure 5c,d,e) confirm the spherical morphology of precursor materials has been reserved after calcination with a particle size ranging within 5–8 μm. The surface feature and size uniformity of the lithiated oxide particles (Figure 5c,d,e) differs from the original oxalate precursors (Figures 2c and 3a). Possible reasons include: (1) the oxalate particles are soft and more prone to morphology change under grinding than hydroxide particles and (2) common phenomena during calcination, such as particle surface features, change with structural water and gas release upon heated precursor decomposition and possible particle agglomeration under high temperature. Nonetheless, the size uniformity of NCM811 cathode particles is still comparable with other studies, indicating uniformly-sized precursors particles (from slug flow synthesis) can tolerate some extent of nonoptimization during calcination and lithiation (to be optimized in the future). The tap density value of 2.4 (±0.1) g mL^–1^ is higher than reported values^23,27^ of 1.95–2.38 g mL^–1^, likely due to improved uniformity of precursor particle size, spherical morphology, and high crystallinity. The chemical composition is confirmed with EDX elemental mapping (Figure 5f,g,h). As a side comment, the calcined particles (Figure 5) do not show an evident core–shell structure as in precursor particles (Figure 4), likely due to the long holding time at a high temperature for calcination/lithiation, which may increase the rate of solid-state diffusion of the metal components and decrease the concentration gradient.

### 3.3 Electrochemical Performance of NCM811

**Figure 6:**
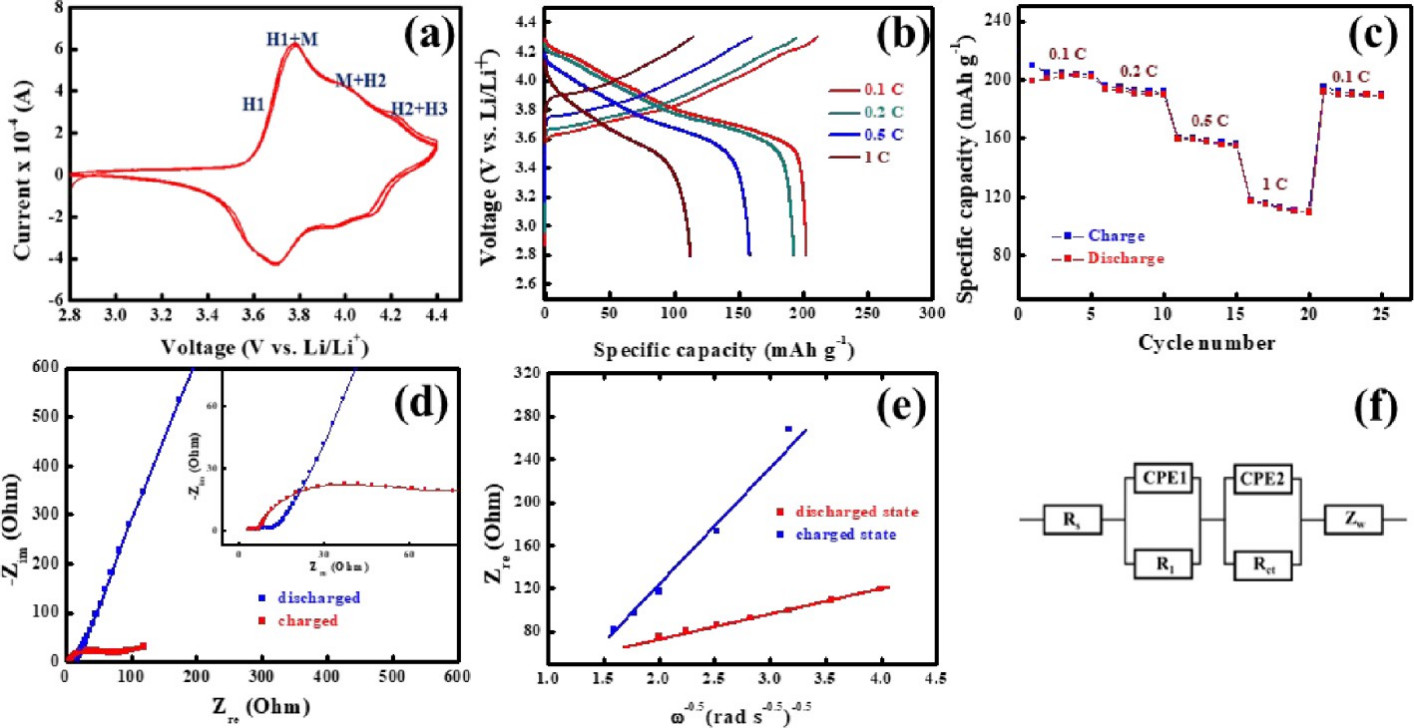
(a) Cyclic voltammetry; (b) charge–discharge profile; (c) rate capability; (d) Nyquist plot; (e) *Z*_re_ vs ω^–0.5^ plot for the NCM811-based LIB half-cell. The high-frequency region of the Nyquist plot is shown in the inset of (d). The equivalent circuit is shown in (f).

NCM811-based LIB half-cells are subjected to various electrochemical experiments to evaluate the Li^+^ storage performance of the cathode. The cyclic voltammetry was performed within the voltage window of 2.8–4.4 V vs Li/Li^+^ at the scan rate of 0.2 mV s^–1^ (Figure 6a). The redox signatures in the cyclic voltammogram indicate the multiple phase change of the cathode during the charging step. Initially, the pristine material was in a hexagonal (H1) phase with high Li content. With the progress of the charging process, the Li^+^ ions started to gradually deintercalate from the structure. The Ni^3+^ ions are also partially oxidized to the Ni^4+^ within the range of 3.75–3.8 V vs Li/Li^+^. The NiO_6_ octahedra related to the Ni^3+^ undergoes Jahn–Teller (J-T) distortion, whereas Ni^4+^-centered NiO_6_ octahedra remain free from J–T distortion. This special ordering of Ni^3+^O_6_ and Ni^4+^O_6_ octahedra leads toward the phase transition from H1 to the monoclinic (M) phase.^58^ Further charging toward 4.0 V vs Li/Li^+^ creates more Li vacancies in the structure and transformation from M to H2, which differs from the initial H1 phase primarily in terms of Li content.^59^ Finally, at the voltage range of >4.2 V, all the transition metals are in the highest valence state and there is a severe volume contraction of the unit cell, causing the phase transformation from H2 to H3.^60^ The charge–discharge experiment (Figure 6b) was performed to evaluate the charge storage performance of the cathode at different C rates within the voltage window of 2.8–4.3 V vs Li/Li^+^. The cathode shows a high specific capacity value of 202 mAh g^–1^ at the 0.1 C rate and retains up to 114 mAh g^–1^ at the higher C rate of 1 C. The much lower capacity at the higher C rate (1 C) is likely due to increased probability of mechanical degradation and parasitic side reaction, hampering Li^+^ diffusion.^61^ From the rate capability plot (Figure 6c), it is found that the cathode retains 98.5% of its initial specific capacity upon decreasing the C rate to 0.1 C again after 20 charge–discharge cycles. The Coulombic efficiency values of the first five cycles at each C rate are shown in Table S2. The efficiency is found to be improved from the first to fifth cycle as the difference between charge and discharge capacity is found to be reduced gradually, which is attributed to the improved Li^+^ diffusion kinetics throughout the cathode.

To analyze the Li^+^ diffusion kinetics of the cathode at charged and discharged states, the impedance analysis was performed at 4.3 V (charged) and 2.8 V (discharged) and the as-obtained Nyquist plots (Figure 6d) are fitted with the suitable equivalent circuit. The Nyquist plot at the charged state is composed of two distinct semicircles at a higher and mid frequency and a straight line at the lower frequency region. The higher frequency and midfrequency semicircles are related to the surface resistance (*R*_l_) and charge transfer resistance (*R*_ct_), respectively. The slope of the lower-frequency straight line, termed as the Warburg component (*Z*_W_), shows the Li^+^ diffusion behavior. In addition to the aforementioned components, the equivalent circuit also contains a solution resistance (*R*_s_) and two constant phase elements (CPE1 and CPE2), which are associated with two of the resistive components with two different time constants (τ = *RC*).^62^ It is observed that the impedance profiles are significantly different at the charged and discharged state of the cathode. The two semicircles are found to be merged, and the Warburg slope becomes steeper in the Nyquist plot of the discharged state. The fitted impedance data for both states are given in Table 2. The *R*_ct_ at the charged state is found to be higher than that of the discharged state. This is ascribed to the higher electron affinity of the high valence transition metals at 4.3 V and deterioration in the integrity between the primary particles of the cathode material.^63^ The Li^+^ diffusion coefficient (*D*_Li+_) in the bulk phase is calculated by the slope of the *Z*_re_ plot (Figure 6e). The equivalent circuit is shown in Figure 6f, and the detail of the calculation is given in the Supporting Information. The high intensity ratio of (003) and (104) in the XRD pattern (1.85) indicates the lesser extent of Ni^2+^/Li^+^ mixing and potentially better Li^+^ diffusion kinetics.^64,65^ It is also observed that the *D*_Li+_ at the discharged state is lower compared to that of the charged state. The primary reason behind the poor Li^+^ diffusion kinetics at the discharged state is that the generation of more Ni^2+^ at the lower voltage region aggravated the degree of Ni/Li mixing in the bulk and caused the blockage of the Li^+^ diffusion channel.^63^

**Table 2:** Data Derived from Impedance Analysis

voltage [V vs Li/Li^+^]	*R*_s_ [Ω]	*R*_ct_ [Ω]	*D*_Li^+^_ [cm^2^ s^–1^]
4.3 (Charged)	3.0	60	9.008 × 10^–9^
2.8 (Discharged)	4.5	11	9.66 × 10^–11^

**Figure 7:**
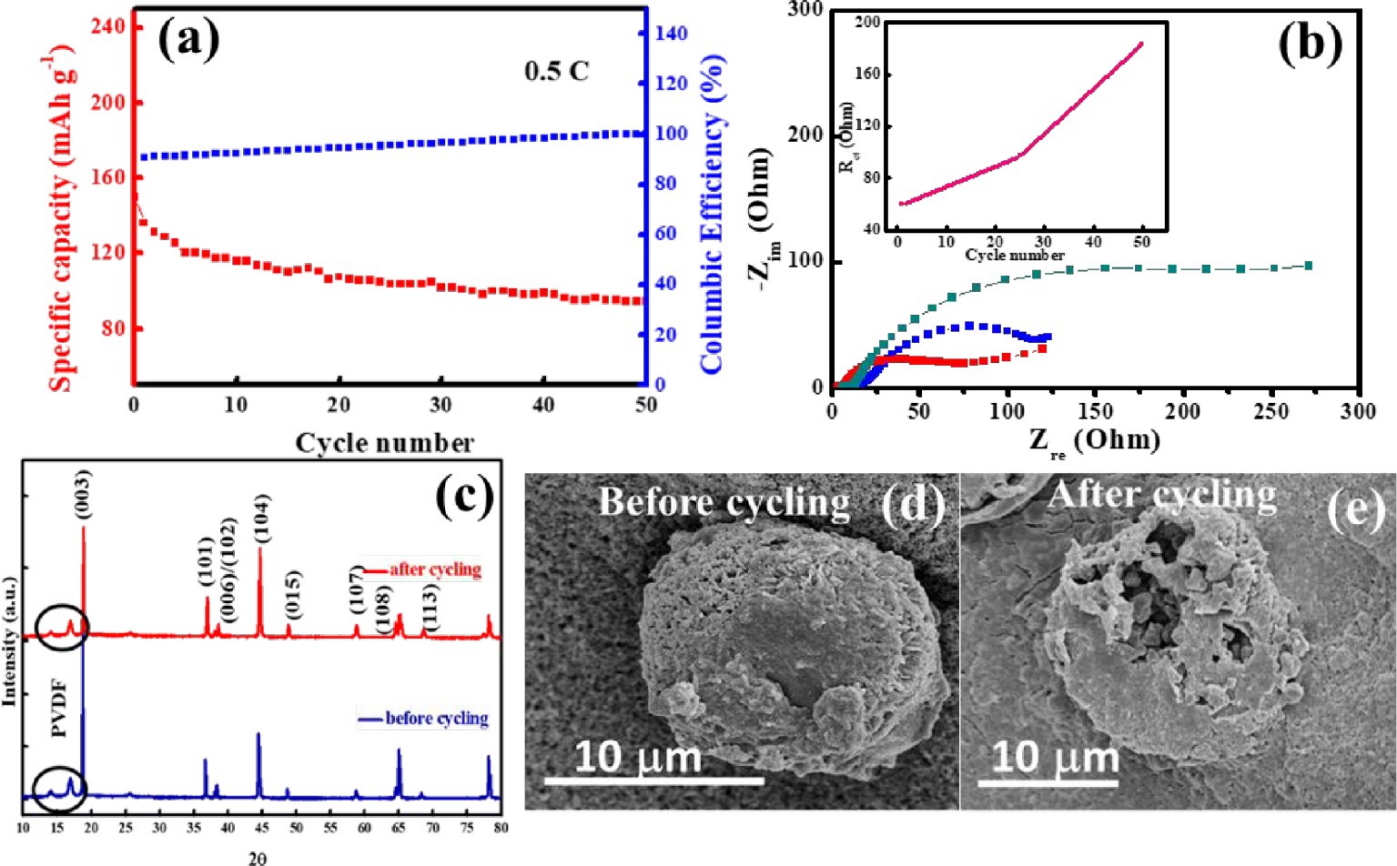
(a) Cycling stability; (b) impedance profiles during cycling of the NCM811-based LIB half-cell. The variation of *R*_ct_ with the cycling is shown in the inset of (b). Postcycling (c) XRD; (d, e) SEM images of NCM cathode materials before and after cycling.

The cycling performance of the cathode is evaluated for 50 charge–discharge cycles at the C rate of 0.5 C within the voltage window of 2.8–4.3 V vs Li/Li^+^ (Figure 7a). 62% of initial specific capacity is found to be retained with a Coulombic efficiency of ∼100% at the end of the cycling test. Impedance analysis was also performed at the various stages of cycling, and the Nyquist plot is shown in Figure 7b. It is observed from the Nyquist plot that *R*_ct_ is abruptly increased from 60 to 190 Ω at the end of the 50 cycles (Figure 7b, inset). Possible reasons for the performance deterioration with cycling include cation mixing and microcracking of cathode particles, as indicated from the postcycling XRD (Figure 7c) and SEM (Figure 7d,e) characterization. The XRD profiles (Figure 7c) show that most of the peaks remain intact after cycling, and the volume expansion of the cathode is slight (from 101.04 to 101.07 Å^3^, Table S1). However, the peak ratio *I*_(003)_/*I*_(104)_ decreases from 1.85 to 1.65, indicating an enhanced degree of Ni/Li mixing after cycling. The NCM811 particles also show microcracking after cycling (Figure 7d,e). Microcracking is not uncommon for high-Ni cathode materials^66^ and is likely due to nonuniform volume contraction and expansion during the charge–discharge cycling.^60,66−68^

### 3.4 Improvement of Performance by Functional Coating

**Figure 8:**
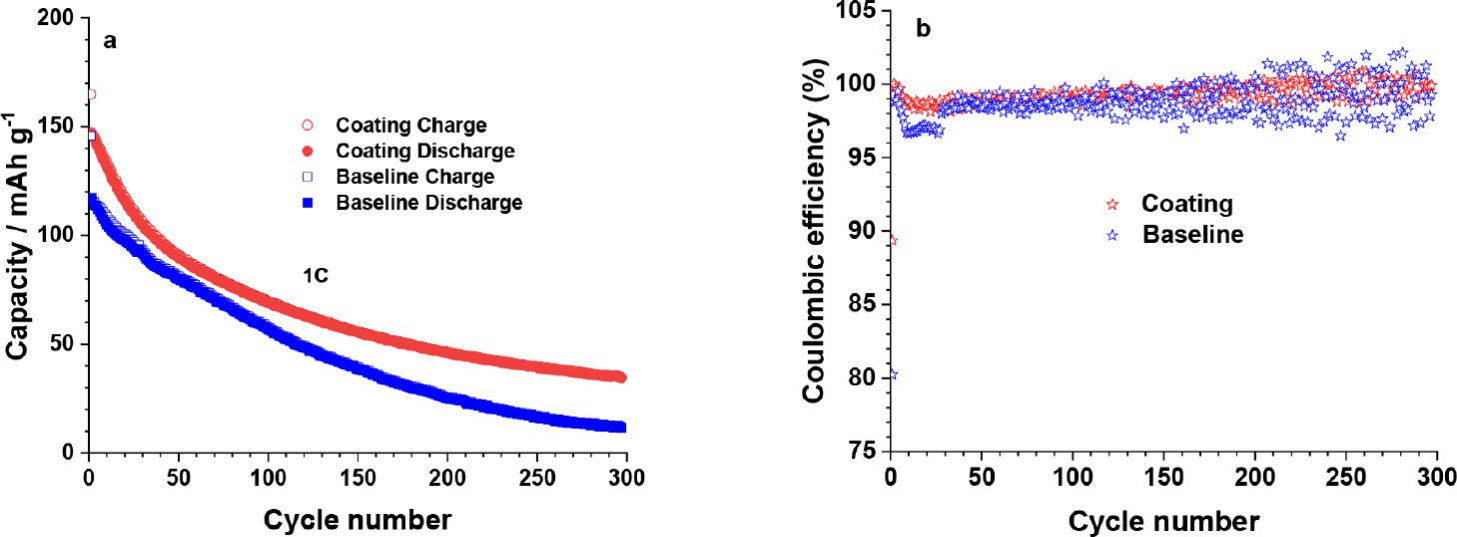
Comparison of cycling stability (a) and Coulombic efficiencies (b) of pristine and coated NCM811 particles in LIB half-cell configuration at 1 C rate after the first three cycles being activated at C/10.

In order to protect the cathode surface from the parasitic side reactions, caused due to the direct contact of the electrolyte, we have also coated NCM811 particles with LiF by treating the powders in a 3 M solution of lithium bis(fluorosulfonyl)imide (LiFSI) in dimethyl carbonate (DMC) followed by postannealing at 60 °C for 24 h.^69^Figure 8a shows higher discharge capacity and cycling stability (300 cycles) of the LiF-coated NCM811 cathode material than uncoated materials at the 1 C rate. In Figure 8b, the initial Coulombic efficiencies of the baseline and coated sample at 1 C are 80.2% and 89.4%, respectively, indicating severe side reactions of the former samples. During the cycling process, the Coulombic efficiencies of the coated sample are higher than those of the baseline sample, particularly during the first 30 cycles, besides more scattered values for the baseline sample. The improvement in the electrochemical performance with coating is consistent with an earlier study^69^ and likely because the cathode particles are protected (to some extent) from side reactions (hampering Li^+^ diffusion) and/or mechanical degradations^68,69^ such as microcracking shown in Figure 7e.

## 4 Conclusion

A novel coprecipitation process has been designed based on multiphase milli-fluidic slug flow for scalable synthesis of uniformly-sized NCM811-oxalate precursor microparticles. Starting from the solution to particles of 5–10 μm in size, the whole precipitation process takes about 10 min, without needing aging that typically takes hours. The oxalate coprecipitation reaction can work at less basic pH (e.g., 8.5) than for common hydroxide reactions (pH = 12). The slug flow synthesis process can be scalable without changing the equipment, by tuning the total running time and slug-to-slug distance. The typical operational issue of fouling is minimized by adding an oil phase between the aqueous slugs (where reaction and crystallization occurs) and tubular reactor walls to prevent their direct contact.

After reaction crystallization, the outcome oxalate precursors can be calcinated and lithiated to oxide microparticles that still preserve the spherical geometry and uniformity, through 2-stage heating at low heating rates (e.g., 0.1 °C/min from room temperature to 500 °C, 0.8 °C/min from 500 to 850 °C). The calcination process for these new NCM811 oxalate precursors will be further improved in the future. The lithiated oxide microparticles demonstrate higher tap density (e.g., 2.4 g mL^–1^ for NCM811) and higher specific capacity (202 mAh g^–1^ at 0.1 C). LiF coating of these pristine NCM811 oxide cathode particles can further improve their Coulombic efficiency and capacity retention.

The phenomena/issue of postcalcination “loss” of core–shell structure and postcycling microcracking will also be topics of future studies, so as to facilitate drawing correlation between the current core–shell structure with NCM performance. In addition, this process will also be adapted to synthesize other composition battery materials at designed scales, such as NCM cathode microparticles with different overall compositions (e.g., NCM111, NCM622, cobalt-free NCM901) and/or doping (Fe or Al) and/or spatial-varying compositions (concentration gradient).

## Supporting Information Available

Fouling at different locations, SEM image, thermal analysis, refinement data for post-cycling XRD, Coulombic efficiency chart, and calculation of Li^+^ diffusion coefficient (PDF)
