# Supplementary material for: Slug Flow Coprecipitation Synthesis of Uniformly-Sized Oxalate Precursor Microparticles for Improved Reproducibility and Tap Density of Li(Ni0.8Co0.1Mn0.1)O2 Cathode Materials
Source: ACS Appl Energy Mater. 2023 Mar 6;6(6):3213–24. doi: 10.1021/acsaem.2c03563 (PMC10064804; doi:10.1021/acsaem.2c03563)
Supplement: Supplementary file 1 — ae2c03563_si_001.pdf [file ae2c03563_si_001.pdf]

Supporting Information

Slug-Flow Co-precipitation Synthesis of Uniformly-Sized Oxalate Precursor Microparticles for Improved Reproducibility and Tap Density of $Li(Ni_{0.8}Co_{0.1}Mn_{0.1})O_2$ Cathode Materials

Mingyao Mou[1], Arjun Patel[1], Sourav Mallick[1], K. Jayanthi[2], Xiao-Guang Sun[2], Mariappan Parans Paranthaman[2], Sophie Kothe[1], Ena Baral[1], Selma Saleh[1], Jethrine H. Mugumya[1], Michael L. Rasche[1], Ram B. Gupta [1], Herman Lopez[3], and Mo Jiang[1,*]

[1]Department of Chemical and Life Science Engineering, Virginia Commonwealth University, Richmond, VA, 23219, USA.
[2]Chemical Sciences Division, Oak Ridge National Laboratory, Oak Ridge, TN, 37831, USA
[3]Ionblox Inc. (*former* Zenlabs Energy Inc.), Fremont, CA, 94538, USA

Corresponding author: Mo Jiang. Email addresses: mjiang3@vcu.edu

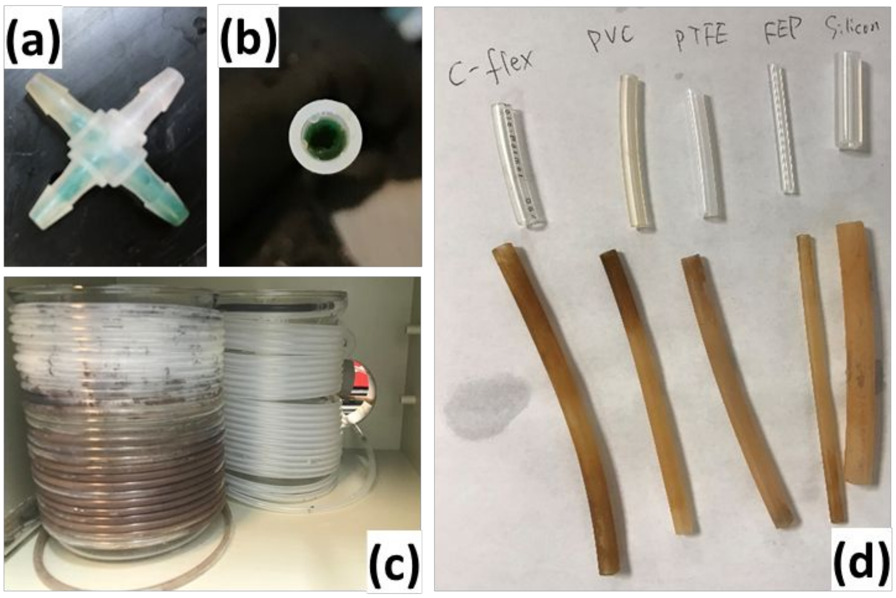

Figure S1. Fouling at different locations: (a) Cross mixer; (c) Main growth zone of a two phase liquid/gas slug flow reactor; (b) Cross-section of the fouling site; and (d) Fouling on different tubing materials, top row is control group and bottom are tubing immersed in the slurry after 30 min, from left to right: C-flex, PVC, PTFE, FEP, Silicon.

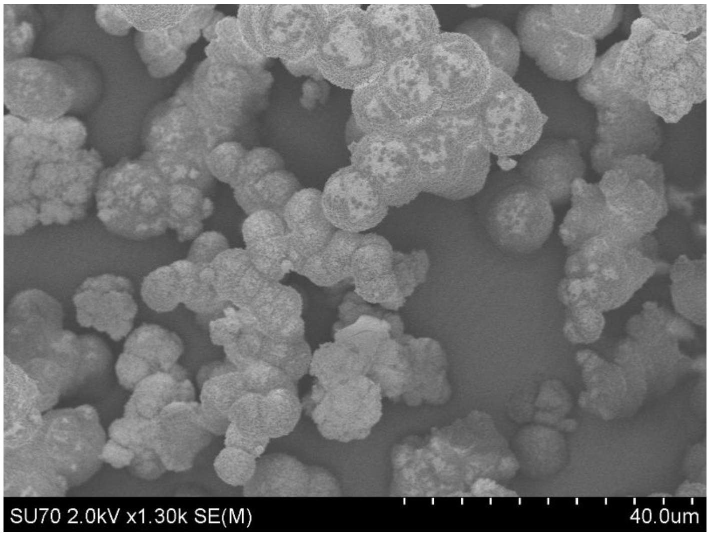

Figure S2. Particle agglomeration was observed at residence time of 2 h in a batch reactor.

Thermal analysis. Both DSC and TGA (Figure S3) showed that 3 steps of thermal activity, 1st stage is the surface water. And the 2nd steps from 150-300°C had ca. 19% weight loss in the TGA and an endothermic peak in DSC. This indicates that the as-synthesized NCM811 oxalate precursor possesses two structural water molecules from calculating the theoretical weight loss value of $N_{0.8}C_{0.1}M_{0.1}C_2O_4\cdot 2H_2O$ (19.7%). Dihydrate is commonly reported for NCM oxalate precursor[1–3]. The decomposition of $N_{0.8}C_{0.1}M_{0.1}C_2O_4$ started around 300°C and completed around 400°C, with obtaining $N_{0.8}C_{0.1}M_{0.1}O$ and release gases such as carbon dioxide or carbon mono-oxide. The as-synthesized precursor had higher thermal stability compared to literature[2,3] (i.e. 230-380°C).) showed that 3 steps of thermal activity, 1st stage is the surface water. And the 2nd steps from 150-300°C had ca. 19% weight loss in the TGA and an endothermic peak in DSC. This indicated that the as-synthesized NCM811 oxalate precursor possesses two structural water molecules from calculating the theoretical weight loss value of $N_{0.8}C_{0.1}M_{0.1}C_2O_4\cdot 2H_2O$ (19.7%). Dihydrate is commonly reported for NCM oxalate precursor[1–3]. The decomposition of $N_{0.8}C_{0.1}M_{0.1}C_2O_4$ started around 300°C and completed around 400°C, with obtaining $N_{0.8}C_{0.1}M_{0.1}O$ and release gases such as carbon dioxide or carbon mono-oxide. The as-synthesized precursor had higher thermal stability compared to literature[2,3] (i.e. 230-380°C).

$$\text{Dehydration: } N_{0.8}C_{0.1}M_{0.1}C_2O_4\cdot 2H_2O \rightarrow N_{0.8}C_{0.1}M_{0.1}C_2O_4 + 2H_2O \qquad (1)$$

$$\text{Decomposition: } N_{0.8}C_{0.1}M_{0.1}C_2O_4 \rightarrow N_{0.8}C_{0.1}M_{0.1}O + \text{gas} \qquad (2)$$

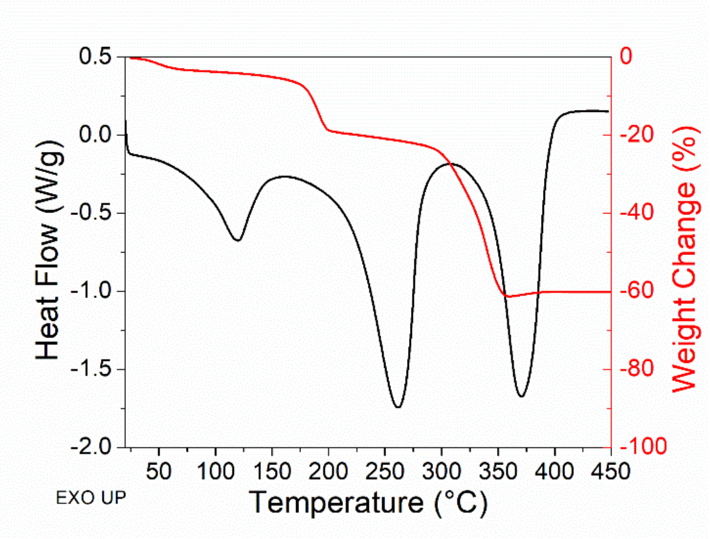

Figure S3. TGA and DSC profile for the NCM811 oxalate precursor.

Table S1. Rietveld refinement-derived crystal parameters of the post-cycling cathode.

$I_{(003)}/I_{(004)}$	a, b (Å)	c (Å)	c/a	Unit cell volume (Å^3)
1.62	2.862	14.238	4.97	101.07

Table S2. Coulumbic efficiency for the first five cycles for NCM-811 at different C-rates.

C-rates	1st	2nd	3rd	4th	5th
0.1	70 %	93.26 %	94.67 %	96.94 %	98.48 %
0.2	96.30 %	97.00%	97.52 %	98 %	98.3 %
0.5	94.8 %	94.85 %	95.3 %	97.2 %	98.5 %
1	79.0 %	79.3 %	79.8 %	80.12 %	80.2 %

Calculation of Li^+ diffusion coefficient. The Li^+ diffusion co-efficient (D_{Li+}) is related with the Warburg component through the following equation:[1]

$$D = R^2T^2(2A^2n^4F^4C^2\sigma^2)^{-1} \qquad \text{(S1)}$$

Where, R =gas constant; T= absolute temperature; A = area of the electrode; N= number of electrons transferred per molecule in the electrochemical reaction; F = Faraday constant; C = concentration of Li^+ ions and σ = Warburg factor. The Warburg factor (σ) can be calculated from the slop of the Z_{re} - $\omega^{-0.5}$ plot using the following relation:

$$Z_{re} = R_s + R_{ct} + \sigma\omega^{-0.5} \qquad \text{(S2)}$$

Reference

(1) Cho, T. H.; Shiosaki, Y.; Noguchi, H. Preparation and Characterization of Layered LiMn1/3Ni1/3Co1/3O2 as a Cathode Material by an Oxalate Co-Precipitation Method. *J. Power Sources* **2006**, *159* (2), 1322–1327.

(2) ZHANG, C. fu; YANG, P.; DAI, X.; XIONG, X.; ZHAN, J.; ZHANG, Y. liang. Synthesis of LiNi1/3Co1/3Mn1/3O2 Cathode Material via Oxalate Precursor. *Trans. Nonferrous Met. Soc. China (English Ed.* **2009**, *19* (3), 635–641.

(3) Nisa, S. S.; Rahmawati, M.; Yudha, C. S.; Nilasary, H.; Nursukatmo, H.; Oktaviano, H. S.; Muzayanha, S. U.; Purwanto, A. A Fast Approach to Obtain Layered Transition-Metal Cathode Material for Rechargeable Batteries. *Batteries* **2022**, *8* (1), 4.
